# Supplementary material for: Therapeutic Prospection of Animal Venoms-Derived Antimicrobial Peptides against Infections by Multidrug-Resistant Acinetobacter baumannii: A Systematic Review of Pre-Clinical Studies
Source: Toxins (Basel). 2023 Apr 3;15(4):268. doi: 10.3390/toxins15040268 (PMC10143903; doi:10.3390/toxins15040268)
Supplement: Supplementary file 1 [file toxins-15-00268-s001.zip › toxins-2264322-supplementary.pdf]

# Supplementary Materials: Therapeutic Prospection of Animal Venoms-Derived Antimicrobial Peptides against Infections by Multidrug-Resistant *Acinetobacter baumannii*: A Systematic Review of Pre-Clinical Studies

**William Gustavo Lima and Maria Elena de Lima**

**Details of the search strategy—24 December 2022**

**Search in Pubmed**

("antimicrobial peptides"[MeSH Terms] OR ("antimicrobial peptides"[Text Word] OR "antimicrobial peptide*"[Text Word])) AND ("toxins, biological"[MeSH Terms] OR ("toxins biological"[Text Word] OR "biological toxins"[Text Word]) OR "Venoms"[MeSH Terms] OR ("Venoms"[Text Word] OR "Venom"[Text Word])) AND ("acinetobacter baumannii"[MeSH Terms] OR ("acinetobacter baumannii"[MeSH Terms] OR ("acinetobacter"[All Fields] AND "baumannii"[All Fields]) OR "acinetobacter baumannii"[All Fields] OR ("acinetobacter calcoaceticus"[MeSH Terms] OR "acinetobacter baumannii"[MeSH Terms])))

**Search in Scopus**

(TITLE-ABS-KEY (“antimicrobial peptides” OR “antimicrobial peptide*”) OR INDEXTERMS (“antimicrobial peptides” OR “antimicrobial peptide*”)) AND (TITLE-ABS-KEY (“toxins, biological” OR “toxins biological” OR “biological toxins” OR “Venoms” OR “Venom”) OR INDEXTERMS (“toxins, biological”

OR "toxins biological" OR "biological toxins" OR "Venoms" OR "Venom")) AND (TITLE-ABS-KEY ("acinetobacter baumannii" OR "acinetobacter calcoaceticus" OR "acinetobacter") OR INDEXTERMS ("acinetobacter baumannii" OR "acinetobacter calcoaceticus" OR "acinetobacter"))

**Web of Science**

ALL=(("antimicrobial peptides" OR "antimicrobial peptide*") AND ("toxins, biological" OR "toxins biological" OR "biological toxins" OR "Venoms" OR "Venom") AND ("acinetobacter baumannii" OR "acinetobacter calcoaceticus" OR "acinetobacter"))

**SciencerDirect**

("antimicrobial peptides") AND ("toxins, biological" OR "Venoms") AND ("acinetobacter baumannii")

**Biblioteca Virtual em Saúde:**

("antimicrobial peptides" OR "Péptidos Antimicrobianos" OR "Peptídeos Antimicrobianos") AND ("toxins, biological" OR "toxinas biológicas" OR "venoms" OR "peçonhas" OR "ponzoñas") AND ("acinetobacter baumannii") *in* **Título, Resumo, Assunto**

## Reference list of included articles


1. Askari, P.; Namaei, M. H.; Ghazvini, K.; Hosseini, M. *In vitro* and *in vivo* toxicity and antibacterial efficacy of melittin against clinical extensively drug-resistant bacteria. *BMC Pharmacol Toxicol*. **2021**, *22*, 42.
2. López-Rojas, R.; Docobo-Pérez, F.; Pachón-Ibáñez, M.E.; de la Torre, B.G.; Fernández-Reyes, M.; March, C.; Bengoechea, J.A.; Andreu, D.; Rivas, L.; Pachón, J. Efficacy of cecropin A-melittin peptides on a sepsis model of infection by pan-resistant *Acinetobacter baumannii*. *Eur J Clin Microbiol Infect Dis*. **2011**, *30*, 1391-1398.
3. Rishi, P.; Vashist, T.; Sharma, A.; Kaur, A.; Kaur, A.; Kaur, N.; Kaur, I.P.; Tewari, R. Efficacy of designer K11 antimicrobial peptide (a hybrid of melittin, cecropin A1 and magainin 2) against Acinetobacter baumannii-infected wounds. *Pathog Dis*. **2018**, *76*, 30184071.
4. Park, H.J.; Kang, H.K.; Park, E.; Kim, M.K.; Park, Y. Bactericidal activities and action mechanism of the novel antimicrobial peptide Hylin a1 and its analog peptides against Acinetobacter baumannii infection. *Eur J Pharm Sci*. **2022**, *175*, 106205.
5. Pashaei, F.; Bevalian, P.; Akbari, R.; Bagheri, K.P. Single dose eradication of extensively drug resistant Acinetobacter spp. In a mouse model of burn infection by melittin antimicrobial peptide. *Microb Pathog*. **2019**, 127, 60-69.
6. Hassan, A.; Ikram, A.; Raza, A.; Saeed, S.; Paracha, R.Z.; Younas, Z.; Khadim, M.T. Therapeutic Potential of Novel Mastoparan-Chitosan Nanoconstructs Against Clinical MDR *Acinetobacter baumannii*: In silico, in vitro and in vivo Studies. *Int J Nanomedicine.* **2021**, *16*, 3755-3773.
7. Brito, J.C.M.; Lima, W.G.; Resende, J.M.; de Assis, D.C.S.; Boff, D.; Cardoso, V.N.; Amaral, F.A.; Souza-Fagundes, E.M.; Fernandes, S.O.A.; de Lima, M.E. Pegylated LyeTx I-b peptide

is effective against carbapenem-resistant Acinetobacter baumannii in an in vivo model of pneumonia and shows reduced toxicity. *Int J Pharm*. **2021**, *609*, 121156.

8. Lima, W.G.; Brito, J.C.M.; de Lima, M.E.; Pizarro, A.C.S.T.; Vianna, M.A.M.M.; de Paiva, M.C.; de Assis, D.C.S.; Cardoso, V.N.; Fernandes, S.O.A. A short synthetic peptide, based on LyeTx I from Lycosa erythrognatha venom, shows potential to treat pneumonia caused by carbapenem-resistant Acinetobacter baumannii without detectable resistance. *J Antibiot (Tokyo)*. **2021**, *74*, 425-434
